# The impact of active sport tourism experiencescape perception on tourists’ psychological recovery: the mediating role of flow experience

**DOI:** 10.3389/fpsyg.2025.1604948

**Published:** 2025-07-02

**Authors:** Hengyi Song, Dongqi Wang, Teng Ma

**Affiliations:** ^1^Graduate School of Education, Shandong Sport University, Jinan, Shandong, China; ^2^School of Sports Leisure, Shandong Sport University, Jinan, Shandong, China

**Keywords:** sports tourism, experiencescape, psychological recovery, flow experience, SEM

## Abstract

**Introduction:**

Based on the SOR framework, this study addresses the widening mental-health challenges—such as anxiety and depression—among urban populations in China amid rapid urbanization. While previous research has highlighted the “therapeutic” function of tourism, few studies have examined the specific mechanism by which the experiencescape of active sport tourism influences psychological recovery via flow experience. To fill this gap, we proposed an “S: active sport tourism experiencescape – O: flow experience – R: psychological recovery” research model.

**Methods:**

Using a structural equation model, we analyzed survey data from 288 active sport tourism participants. The experiencescape was categorized into six dimensions: social, functional, natural, cultural, sensory, and hotel culture.

**Results:**

The results indicate that the active sport tourism experiencescape exerts significant positive effects on both flow experience and psychological recovery, with flow experience partially mediating the effect on psychological recovery. The six experiencescape dimensions differ in their impacts: social, natural, sensory, and hotel culture dimensions directly and positively influence psychological recovery, whereas functional and cultural dimensions influence psychological recovery only indirectly through flow experience. Flow experience partially mediates the effects of social, natural, sensory, and hotel culture dimensions on psychological recovery, but fully mediates the effects of the functional and cultural dimensions.

**Discussion:**

This study systematically unpacks the mechanisms of the six-dimensional experiencescape in active sport tourism, enriching the theoretical understanding of sport tourism, flow experience, and psychological recovery. The findings offer practical guidance for destination managers to optimize experiencescape elements and enhance visitors’ mental and physical well-being.

## 1 Introduction

Against the backdrop of rapid urbanization in China, the continued population growth in mega- and super-cities, combined with an accelerated pace of modern life, has exposed urban residents to unprecedented mental-health challenges—such as anxiety, depression, and insomnia—which are becoming increasingly prevalent (Xiao et al., 2024). Tourism, as an effective means of “therapeutic” intervention, has received growing scholarly attention for its role in enhancing individual mental health. Empirical studies indicate that interaction between individuals and their environments during travel—particularly within natural or cultural settings characterized by restorative attributes—can facilitate psychological recovery, manifesting as stress reduction, attention restoration, and mood improvement (Yang and Sha, 2024; Lehto, 2013; Qiu et al., 2021a; Xi et al., 2021; Huang et al., 2022). Sport tourism refers to leisure-based travel in which individuals temporarily leave their home communities to participate in or spectate sporting activities, or to express admiration for sport-related attractions. Among the classification schemes for sport tourism, Gibson’s tripartite typology is most widely adopted, distinguishing Active Sport Tourism, Event Sport Tourism, and Nostalgia Sport Tourism (Gibson, 1998). Active Sport Tourism involves the highest level of physical engagement and is emblematic of sport tourism; it typically entails travelers’ participation in specific forms of physical activity—such as mountaineering, skiing, hiking, or marathon running (Xie et al., 2021). Compared to other forms of sport tourism, Active Sport Tourism demands greater physical investment and places stronger emphasis on experiential engagement and interaction. This tourism mode offers travelers unique opportunities for deep contact with nature, cross-cultural exchange, and social connection, thereby serving as a vital pathway for improving psychological well-being and facilitating mind–body recuperation (Su et al., 2022; Xu and Chen, 2024; Wang and Wen, 2024). Psychological recovery refers to changes in an individual’s psycho-physiological state arising from interaction with the environment, chiefly reflected in reduced stress, diminished fatigue, restored attention, and improved mood (Buckley and Westaway, 2020; Huang et al., 2022). The concept of the tourism experiencescape has evolved from service-scape theory, extending its application from indoor service environments to tourism destinations. It has been shown to influence travelers’ flow experience, subjective well-being, and psycho-physiological health (Tasci and Pizam, 2020), and has recently been employed to elucidate the effects of various tourism elements on positive affect (Zhang and Xu, 2019), place attachment (Zhang and Xu, 2023), and flow experience (Huang, 2024). As a key psychological mechanism in Active Sport Tourism, flow experience enables travelers to achieve a state of intense concentration and enjoyment during activities, thereby exerting beneficial effects on mental health (Da Silva DeMatos et al., 2021). However, there is a paucity of systematic research on the specific mechanisms by which experiencescapes in Active Sport Tourism influence psychological recovery. Most existing studies focus on general tourism contexts and have not fully elucidated the recovery mechanisms in this unique form of tourism, which deeply integrates physical activity with emotional experience. In light of this gap, the present study adopts the Stimulus–Organism–Response (SOR) framework to propose an “S: Active Sport Tourism experiencescape – O: Flow experience – R: Psychological recovery” model, aiming to investigate how different dimensions of the experiencescape influence travelers’ psychological recovery via flow experience.

## 2 Literature review

### 2.1 Stimulus: active sport tourism experiencescape

The concept of the active sport tourism experiencescape is derived from a systematic review of related concepts, including service scape, experiencescape, and tourism experiencescape, and further refined by integrating the unique characteristics and inherent essence of active sport tourism.

The development of the concept of servicescape laid the foundation for the emergence of the experiencescape concept. A scape refers to the way people perceive and imagine their surrounding environment and is often used to describe diverse environmental characteristics across industries. Different perceptions or imaginations exist in certain forms, evoking positive or negative emotions toward these scapes (Appadurai, 1996). Kotler (1973) first introduced the term “atmospherics” to explain the specific emotional responses consumers experience in a consumption environment. These responses are shaped by visual, auditory, olfactory, and tactile stimuli, which influence customers’ emotions and cognition, thereby enhancing their purchase intentions. Bitner (1992) introduced the concept of “scape” into service research and proposed the term “servicescape,” referring to the various physical environmental elements of a service location that are deliberately designed and managed. The elements of servicescape include three dimensions: ambiance, spatial layout and functionality, as well as signs, symbols, and artifacts. While “atmosphere” tends to focus more on the individual level, “servicescape” emphasizes the overall dimensions and elements of the environment and is used to evaluate the overall impact of the service environment (Venkatraman and Nelson, 2008). Consequently, the concept of “servicescape” has attracted attention from scholars across various fields and, with the development of related research, has been applied to diverse products and services.

The concept of experiencescape originated from the transition from a service economy to an experience economy, where consumers’ subjective psychological perceptions increasingly became the focus of the service industry (Li and Zhang, 2022). The significance of experiencescape lies in enhancing consumers’ experiences by managing external environmental factors (Chang, 2018). O'Dell (2005) first introduced the concept of “experiencescape,” which refers to spaces for various types of human activities, interactions, pleasures, entertainment, and enjoyment. Subsequently, the concept of experiencescape gained increasing attention and development among scholars. Willson and McIntosh (2007) refined the concept, defining experiencescape as a space imbued with emotion, focus, engagement, and personal meaning. Pizam and Tasci (2019), based on stakeholder theory, suggested that experiencescape refers to the sensory, natural, social, cultural, functional, and hospitality culture stimuli within the product or service environment. These elements collectively shape the experiences of consumers, employees, and other stakeholders. Kandampully et al. (2022) synthesized the literature on servicescape, service quality, customer experience, and experiencescape, identifying technology as a key component of the experiencescape concept. The difference between experiencescape and servicescape lies in the former’s stronger emphasis on the embodiment, interactivity, and engagement of the consumption environment, as well as its value in the generation of consumer emotions and personal meaning (Li and Zhang, 2022).

Tourism experiencescape is considered a comprehensive perception of both tangible and intangible environmental elements that tourists encounter across the entire destination, rather than within specific service venues of the destination (Zhang and Xu, 2019). In recent years, the concept of experiencescape has been increasingly applied in the tourism sector, including rural tourism (Liu and Lin, 2024), red tourism (Li and Zhang, 2022), coastal tourism (Hu and Chen, 2024), Hanfu tourism (Zong et al., 2023), military tourism (Chen et al., 2024), ski tourism (McLeay et al., 2019), mountain tourism (Vespestad and Hansen, 2020), and natural tourism (Fossgard and Fredman, 2019). Regarding the dimensional division of tourism experiencescapes, Zhang and Xu (2023) proposed four dimensions: physical, natural, social, and social-symbolic, making it one of the most frequently-used m frameworks for dimensional division. Additionally, some scholars have employed qualitative analysis to identify the dimensions of experiencescapes based on research objectives and specific contexts. For instance, rural tourism experiencescape includes five dimensions: natural, atmosphere, hospitality culture, experiential activities, and agri-creative products (Liu and Lin, 2024); Hanfu tourism experiencescape comprises seven dimensions: natural, social, cultural, functional, sensory, hospitality culture, and technological dimensions (Zong et al., 2023); red tourism experiencescape includes thematic atmosphere elements (such as architectural appearance, interior design, and thematic displays) and social participation elements (such as storytelling, interpersonal interaction, and activity engagement) (Li and Zhang, 2022).

Although the aforementioned theories can offer insights for the concept of active sport tourism experiencescape, there are significant differences between the experiencescapes of active sport tourism and general tourism on multiple levels. Firstly, tourism experiencescape is often applied in urban tourism destinations, whereas active sport tourism typically takes place in areas with rich natural environments, where tourists deeply interact with nature through activities such as hiking and mountaineering. Secondly, tourism experiencescape often neglects local ambiance and cultural elements, overly focusing on the physical dimension while lacking experiential and participatory aspects. In contrast, active sport tourism emphasizes deep interaction and cooperation among tourists, local residents, and service providers (Konu et al., 2011), and places greater emphasis on cultural heritage and experiential activities. Therefore, directly applying the concept of tourism experiencescape to active sport tourism fails to capture its participatory and experiential essence. Additionally, active sport tourism, with its high degree of participation and experiential engagement, offers richer sensory stimulation and a deeper and more intense embodied experience compared to general tourism (Xie et al., 2021). Experiencescape places greater emphasis on the embodied nature, interactivity, and participation of consumption spaces, as well as their value in the generation of emotional and personal meanings for consumers, providing a theoretical foundation for the proposal of active sport tourism experiencescape. Finally, based on the dimensional classifications of experiencescape (Pizam and Tasci, 2019) and tourism experiencescape (Zong et al., 2023), active sport tourism experiencescape refers to the context within the entire destination and its sports activities that comprehensively shapes and enhances tourists’ physical and mental experiences. It specifically includes six dimensions: social, functional, natural, cultural, sensory, and hospitality culture.

The social dimension refers to the process and experience of tourists interacting with others, establishing social connections, and participating in group activities during their journey. The functional dimension pertains to the functionality of infrastructure and services in sports activities. The natural dimension encompasses tourists’ experiences in natural environments, including the aesthetic appeal of natural landscapes, architectural settings with natural elements, and the uniqueness of ecosystems. The cultural dimension within the active sport tourism experiencescape refers to the cultural experiences of tourists during their journey, including exposure to local culture, visits to historical heritage sites, participation in traditional activities, and cultural exchange processes. The sensory dimension in the active sport tourism experiencescape denotes the experiences tourists gain through sensory stimulation, which involve a combination of visual, auditory, olfactory, gustatory, and tactile sensations. The hospitality culture dimension in the active sport tourism experiencescape refers to the hotel services, design styles, and overall experiences that tourists encounter during their stay. Therefore, the active sport tourism experiencescape builds upon the social, functional, natural, cultural, sensory, and hospitality culture dimensions of the tourism experiencescape. It further integrates the unique participatory, experiential, and interactive aspects of sports activities, showcasing diversity while highlighting the distinctiveness of active sport tourism.

### 2.2 Organism: flow experience

The concept of flow experience, first introduced by American psychologist Csikszentmihalyi (1975), refers to a psychological state characterized by intense focus, immersion, and enjoyment during an activity. In this state, individuals become fully engaged in the current activity, perceive a slowing of time, and experience profound satisfaction and a sense of control, resulting in strong intrinsic motivation. This psychological state differs from traditional motivation theories as it is not triggered by external incentives but originates from the intrinsic rewards of the activity itself (Chen, 2014). Therefore, flow experience is commonly observed in activities that are both challenging and fully engage an individual’s abilities, such as artistic creation, sports, and work tasks (Csikszentmihalyi, 1990). In active sport tourism, the challenging and engaging nature of activities makes it easier for tourists to enter a state of flow, enabling them to gain profound emotional experiences and psychological satisfaction during the activities (Nakamura and Csikszentmihalyi, 2002).

Flow experience has a unique advantage in explaining tourists’ emotional and behavioral responses, and as a result, it has been widely applied in tourism research. For example, Liang and Liu (2023) developed a relationship model based on the S-O-R theory, exploring the connection between tourists’ photography motivations, flow experience, and post-trip behavioral intentions. Kim and Thapa (2018) explored the relationship between the factors influencing tourists’ flow experience and satisfaction. Wang et al. (2024) revealed the mechanism by which flow experience, as an intrinsic motivator, influences adventure behavior intentions among mountain explorers.

### 2.3 Response: psychological recovery

The concept of psychological recovery originates from the broad definition of recovery, initially used to explain recovery at the physiological level. As research progressed, it gradually found widespread application in fields such as environmental psychology, management, and tourism studies. In the field of psychology, the focus of recovery has expanded from physiological recovery to psychological recovery, referring to the process through which an individual restores their psychological state to a balanced or positive condition after experiencing mental fatigue, stress, or emotional exhaustion, often through certain behaviors, environments, or activities (Sonnentag and Fritz, 2007). In recent years, psychological recovery in tourism research has mainly focused on the improvement of individuals’ psychological states after engaging with natural environments, participating in leisure activities, or undergoing tourism experiences. It emphasizes achieving recovery in psychological aspects such as stress, emotions, mental well-being, and attention through the interaction between the environment and activities (Liu and Lin, 2024; Xi et al., 2021; Xiao et al., 2024; Xu and Chen, 2024).

The determinants of psychological recovery are multifaceted, with restorative environments being a key factor influencing psychological recovery. Natural environments, with their unique restorative characteristics, offer quietness, safety, aesthetic appeal, and spaces that distance individuals from everyday stressors, allowing them to temporarily escape cognitive overload and, in turn, restore psychological energy (Zhou et al., 2020). Social interaction and cultural atmosphere, through positive social support and engagement, can provide emotional support, sense of belonging, and identification (Cheng et al., 2019; Zhang et al., 2021). Moreover, individual behaviors and their psychological mechanisms are also important drivers of psychological recovery. Active participation in leisure activities, physical exercise, or tourism experiences can enhance physical vitality and psychological enjoyment, thereby facilitating the process of psychological recovery (Hartig et al., 2003). Flow experience, as a psychological state of high concentration and immersion, can significantly enhance the effectiveness of psychological recovery by providing deep psychological satisfaction and intrinsic motivation (Csikszentmihalyi, 1990). Although there is extensive research on psychological recovery, limited scholars have explored psychological recovery within the context of active sport tourism experiencescapes. Therefore, based on experiencescape theory and flow experience theory, this study explores the alleviation of stress and fatigue, the restoration of attention, and the adjustment and improvement of emotions experienced by tourists during their travel.

## 3 Hypotheses and theoretical framework

### 3.1 The impact of experiencescape on psychological recovery

The natural dimension of experiencescapes is widely recognized for its positive impact on mental health, particularly in promoting psychological recovery. The restorative characteristics of natural environments, such as diversity, tranquility, and their distance from urban areas, help tourists temporarily escape from daily mental burdens, enhance individual focus and cognitive functions, and provide a space and time for resetting their mental state, thus having a profound impact on psychological recovery (Kjellgren and Buhrkall, 2010; Liu and Lin, 2024; Zheng et al., 2017). Meanwhile, in active sport tourism, the higher level of physical participation of tourists in such contexts brings strong sensory stimulation. The experiences derived from sensory stimuli, including visual, auditory, olfactory, gustatory, and tactile sensations, not only alleviate psychological fatigue caused by daily life stress but also stimulate positive emotional responses such as joy, calmness, and satisfaction, all of which directly promote psychological recovery (Qiu et al., 2021b; Su et al., 2022; Xu and Chen, 2024; Zhou et al., 2023).

The hotel culture dimension of experiencescapes has long attracted scholarly attention, especially in the context of active sport tourism, where the quality of sleep and adequate rest are key factors influencing the tourism experience. The quality of service and facility conditions in hotels can directly affect tourists’ comfort and satisfaction. High-quality service and a comfortable environment can effectively relieve fatigue, improve sleep quality, and thus enhance the psychological recovery effect (Durna et al., 2015; Mao et al., 2018; Yang et al., 2022). Furthermore, the functional dimension of experiencescapes, by providing high-quality facilities, reasonable activity arrangements, and safety assurances for sports activities, can directly influence tourists’ comfort and satisfaction during the tourism process (Tasci and Pizam, 2020). The social dimension of experiencescapes can provide emotional support, a sense of belonging, and identity, which is of unique importance in promoting tourists’ psychological recovery (Huang et al., 2022). Social interactions can promote positive social relationships among tourists, residents and staff, reduce feelings of loneliness, and enhance individual pleasure and well-being (Line and Hanks, 2019; Zhang and Xu, 2023). Culture is a primary dimension influencing human behavior, as it defines individuals’ cognitive, emotional, and behavioral responses to environmental stimuli (Hofstede, 2001; Rokeach, 1973). Different tourists may actively prefer places with symbols and artifacts that reflect their socio-cultural identity, as well as individuals whose appearance and behavior resemble their own. Therefore, the cultural dimension significantly influences tourists’ psychological states and the overall quality of their experience (Tasci and Pizam, 2020).

In summary, the active sport tourism experiencescape, as a second-order construct, encompasses multiple experiential dimensions, including social, functional, natural, cultural, sensory, and hospitality culture. These dimensions interact to form a comprehensive and complex systemic recovery environment. By providing diverse sensory and cognitive stimuli, they meet the physiological, psychological, and social needs of individuals, helping them relieve the stress and anxiety of daily life, enter a state of deep relaxation and pleasure, and achieve comprehensive psychological recovery and overall health improvement through a holistic experience. Therefore, the following hypotheses are proposed:

*H1a*: Active sport tourism experiencescapes can positively influence psychological recovery.

*H1b*: Different dimensions of active sport tourism experiencescapes can significantly positively influence psychological recovery.

### 3.2 The mediating role of flow experience

The intrinsic relationship between flow experience and tourism has long been a subject of interest in tourism research. In recent years, an increasing number of scholars have empirically examined the correlation between service environments and flow experience. The results indicate that factors such as nature, physical environment, and social elements affect flow experience, thereby influencing tourists’ emotions and satisfaction (Huang, 2024; Lee et al., 2012).

The concept of experiencescape developed from the servicescape, and its multidimensional experiential elements exert a combined influence on tourists’ psychological and behavioral responses (Tasci and Pizam, 2020). The natural dimension of the experiencescape has a unique advantage in promoting flow experience. The tranquility and diversity of natural environments provide tourists with a space away from daily stress, making it easier for them to focus on the current activity, thereby positively influencing the emergence of flow experience (Huang, 2024). At the same time, in the context of active sports tourism, multisensory stimulation—particularly the combined effects of visual, auditory, and tactile stimuli—directly enhances tourists’ emotional responses and psychological pleasure. Rich sensory experiences not only increase tourists’ sense of presence but also enhance their focus, making it easier for them to enter a flow state (Lu et al., 2022; Wöran and Arnberger, 2012). In addition, social interactions in active sports tourism not only provide emotional support to tourists but also strengthen their sense of belonging and identification by fostering positive social relationships, thereby enhancing their perception of the surrounding environment and promoting emotional and cognitive connections with others (Line and Hanks, 2019). Whether interacting with other tourists or communicating with local residents and service staff, these positive social relationships enhance tourists’ focus and continuity in sports activities, thereby strengthening the flow experience (Huang, 2024; Weng and Li, 2020). The cultural dimension of the experiencescape further promotes the emergence of flow experience by enriching the content of experiences and enhancing emotional resonance. When tourists engage in activities with cultural significance, they are more likely to form emotional connections and a sense of identification. This sense of identification enhances their focus and immersion, thereby facilitating the occurrence of flow experience (Huang, 2024; Tasci and Pizam, 2020).

The key to flow experience lies in the balance between an individual’s abilities and the demands of the task when facing challenges. The functional dimension of the experiencescape provides appropriate challenges and support through the rational design of the external environment and high-quality hardware facilities, allowing tourists to maintain high focus and making it easier for them to enter a flow state (Chen et al., 2023; Wu and Liang, 2011). At the same time, high-quality accommodation and services help tourists to rest fully after a day’s activities, providing a solid psychological and physiological foundation for subsequent activities. The hospitality cultural dimension creates a comfortable and safe resting environment, helping tourists maintain a positive psychological state, making it easier for them to focus and enter a flow state during activities (Da Silva DeMatos et al., 2021). In summary, the active sports tourism experiencescape, as a second-order construct, has a significant positive impact on flow experience through its dimensions of social, functional, natural, cultural, sensory, and hospitality culture. These dimensions, through different mechanisms, jointly create an ideal psychological environment for tourists, promoting the formation of a flow state. Therefore, the following hypotheses are proposed:

*H2a*: The active sports tourism experiencescape can significantly positively influence flow experience.

*H2b*: Different dimensions of the active sports tourism experiencescape can significantly positively influence flow experience.

Flow experience enhances an individual’s focus and psychological engagement, effectively isolating external distractions and negative emotions, allowing the individual to immerse fully in the current activity (Csikszentmihalyi, 1990). This deep psychological engagement, marked by heightened focus and altered time perception, leads individuals to experience the swift passage of time and a pleasurable sense of immersion during the activity. This immersion not only alleviates stress and anxiety from daily life but also enhances the individual’s sense of satisfaction and achievement, positively promoting psychological recovery (Chen, 2014; Da Silva DeMatos et al., 2021). Furthermore, when an individual’s skill level matches the challenge level of the activity, this state is often accompanied by positive emotional experiences and the restoration of psychological energy (Cai et al., 2018). Therefore, flow experience not only has a positive effect on the activity itself but also enhances the individual’s emotional regulation abilities, promoting overall psychological recovery and improving their health levels. Therefore, the following hypothesis is proposed:

*H3*: Flow experience can positively influence psychological recovery.

Based on the above analysis, the active sports tourism experiencescape, through its multidimensional characteristics (such as social, functional, natural, cultural, sensory, and hospitality culture), provides rich sensory stimuli and emotional resonance, thereby laying the foundation for psychological recovery. Psychological recovery is not solely a direct result of the experiencescape. Under each dimension of the active sports tourism experiencescape, flow experience can transform the positive elements of the experiencescape into a promoting factor for psychological recovery. Flow experience, as the key mechanism in this process, connects the multidimensional experiencescape with psychological recovery, acting as a mediator that transforms the positive effects of the experiencescape into psychological recovery outcomes. Therefore, the following hypotheses are proposed:

*H4a*: Flow experience plays a significant mediating role in the effect of the active sports tourism experiencescape on psychological recovery.

*H4b*: Flow experience plays a significant mediating role in the effect of different dimensions of the active sports tourism experiencescape on psychological recovery.

### 3.3 Model construction based on the S-O-R theory

Russell (1974) proposed the “Stimulus-Organism-Response” (S-O-R) theoretical framework to describe and explain how external stimuli lead to behavioral responses through psychological and emotional processes within the organism. Stimulus: Refers to the factors in the external environment that can trigger changes in an individual’s psychological and behavioral responses. These factors can include elements from the physical, social, or cultural environment. Organism: Refers to the psychological and emotional processes within the individual, including cognition, emotion, attitudes, etc. At this stage, the individual processes and responds to stimuli, forming specific psychological states. Response: Refers to the behavioral or attitudinal responses exhibited by the individual after being stimulated and processing it through psychological and emotional responses (Belk, 1975).

**Figure 1:**
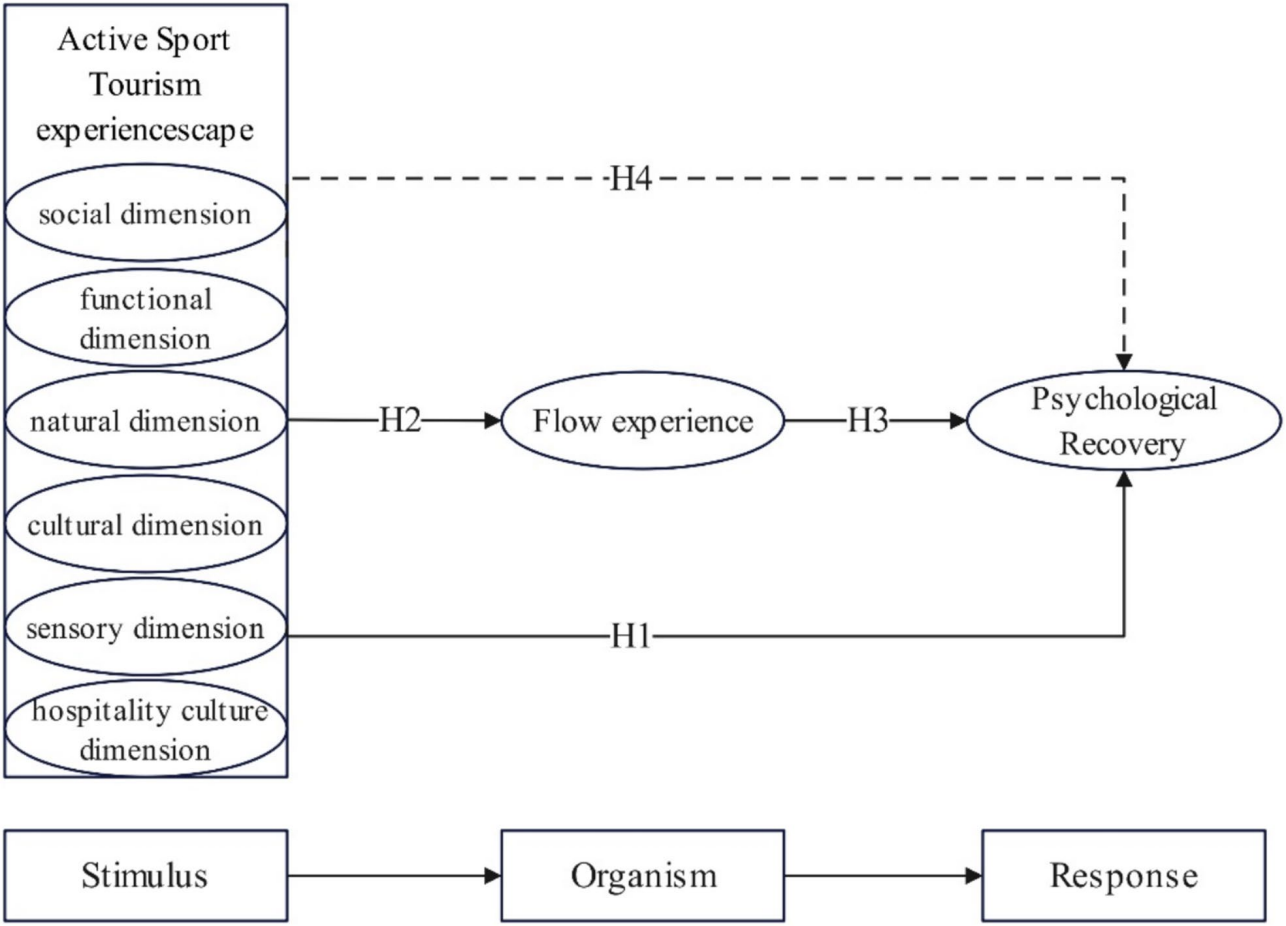
Conceptual model.

In the field of tourism, many scholars have applied the S-O-R theoretical framework to empirical research (Huang, 2024; Jang et al., 2020; Liang and Liu, 2023; Xu and Chen, 2024). In the context of active sports tourism, the specific environments and conditions in sports activities, such as the natural environment, social interactions, and cultural atmosphere, serve as external stimuli that directly affect tourists’ psychological and emotional states. Flow experience is a core psychological process in active sports tourism. It describes the mental state of being fully absorbed and highly enjoying the current activity, acting as a key bridge between the experiencescape and psychological recovery (Csikszentmihalyi, 1975; Kim and Thapa, 2018; Santhanam et al., 2016). Through this mediating role, flow experience explains how tourists, in the context of active sports tourism, achieve psychological recovery by being fully immersed and focused. Therefore, this study proposes that the active sports tourism experiencescape, as an external environmental stimulus, will evoke flow experience in tourists, ultimately affecting their psychological recovery. In summary, this study proposes a conceptual model aimed at examining the relationship between perceptions of the active sports tourism experiencescape, flow experience, and psychological recovery, as shown in Figure 1.

## 4 Methods

### 4.1 Measures

This study integrates measurement scales from authoritative domestic and international literature, and, in consultation with experts in the field of sports tourism in China, identifies the measurement dimensions and items for the latent variables of active sports tourism experience, flow experience, and psychological recovery. The active sports tourism experience scale is divided into six sub-dimensions: social, functional, natural, cultural, sensory, and hospitality culture. Since the active sports tourism experience involves the influence of the sports tourism context, this study primarily references the experiencescape concept and scale proposed by Pizam and Tasci (2019). Additionally, relevant research literature on experiencescape was consulted, and reviews from platforms such as Xiaohongshu and Ctrip were referenced to ensure that the measurement items accurately assess the dimensions of the experiencescape and align with the context of active sports tourism. The cultural, social, and hospitality culture dimensions were mainly based on the research by Zong et al. (2023), resulting in 5 items for the Cultural dimension, 4 items for the Social dimension, and 4 items for the Hospitality Culture dimension. Pizam and Tasci (2019) suggested that design, layout, space, and signage all constitute the overall function of a space. Thus, the Functional dimension was adapted from the physical dimension of service settings, with reference to Huang (2024), resulting in 5 items. The Natural dimension was primarily based on the study by Liu and Lin (2024), resulting in 5 items. The Sensory dimension was mainly adapted from the scale developed by Pizam and Tasci (2019), tailored to the characteristics of Active Sports Tourism, resulting in 6 items. The Flow Experience dimension was primarily based on the study by Huang (2024), resulting in 4 items. The Psychological Recovery dimension was primarily based on the studies by Xiao et al. (2024) and Liu and Lin (2024), resulting in 5 items. All items in the aforementioned scales were measured using a Likert 5-point scale, with 1 indicating strong disagreement and 5 indicating strong agreement. For the English version of the scales, a translation-back-translation procedure was used to convert them into Chinese. At the same time, anonymous questionnaire completion, clarification that the data would be used for scientific research only, randomizing the order of scale items, and including reverse-scored items were employed to mitigate common method bias.

### 4.2 Procedure and participants

The data collection for this study was conducted in two stages. The first stage was a pre-survey, where 124 questionnaires were distributed online through WeChat groups related to active sports tourism from July 17 to 22, 2024. A random red envelope reward of 2–8 RMB was offered to participants who completed valid questionnaires. A total of 85 valid questionnaires were collected, resulting in a response rate of 68.5%. Reliability analysis and exploratory factor analysis revealed that some items had cross-loadings or loadings below the acceptable threshold. Based on these results, items were either deleted or revised to form the final active sports tourism experiencescape scale used in the formal survey. The second stage was the formal survey. Initially, from July 30 to August 5, 2024, 203 questionnaires were distributed to the target population through the Wenjuanxing platform. Subsequently, an additional 105 questionnaires were distributed online through WeChat groups related to active sports tourism from August 8 to 14, 2024. A total of 305 questionnaires were collected from both distributions, of which 288 were valid, resulting in a valid response rate of 94.43%.

Sample characteristics for the formal survey: The most popular sports tourism activity among the sample was hiking, accounting for 41.7%, followed by skiing (16.0%) and mountaineering (26.0%). Other activities, such as diving, surfing, marathon, and rafting, together accounted for 16.4%. The sample was predominantly male, with 57.3% of participants being male and 42.7% female. In terms of age, 97.2% of respondents were under 50 years old. The largest group was aged 31–40 years, representing 49.3%, followed by 26–30 years (18.8%) and 41–50 years (18.4%). The education level of the respondents was generally high, with 68.1% holding a bachelor’s degree and 25.0% having graduate degrees or higher. In terms of monthly income, the respondents were predominantly from higher income brackets, with 38.5% earning between 5,001 and 10,000 RMB and 49.3% earning more than 10,000 RMB (see Table 1).

**Table 1:** Demographic characteristics (*N* = 288).

Category	Options	*n*	%
Gender	Female	123	42.7
	Male	165	57.3
Age	18–25	31	10.8
	26–30	54	18.8
	31–40	142	49.3
	41–50	53	18.4
	Above 51	8	2.7
Education	High school or below	5	1.7
	Junior college	15	5.2
	Undergraduate	196	68.1
	Post graduate or above	72	25
Monthly income (RMB)	<2000	16	5.6
	2001–5,000	19	6.6
	5,001–10,000	111	38.5
	>10,000	142	49.3
Project	Ski	46	16
	Hiking	120	41.7
	Mountaineering	75	26
	Marathon	16	5.6
	Others (such as diving, surfing, drifting, etc.)	31	10.8

### 4.3 Data analysis methods

The data analysis was carried out in three stages. First, confirmatory factor analysis was performed using SPSS 27.0 and AMOS 24.0 to assess the reliability and validity of the scales. Second, AMOS 24.0 was used to test the goodness-of-fit of the structural model and the direct hypotheses. Finally, the bias-corrected percentile Bootstrap method (with 5,000 resamples) was used to test the mediation effects.

## 5 Results

### 5.1 Common method bias and data normality tests

The Harman single-factor test based on CFA showed the following results: *χ*^2^ = 1656.728, *df* = 527, *χ*^2^/*df* = 3.144, CFI = 0.689, TLI = 0.669, SRMR = 0.086, RMSEA = 0.083. None of the indices met the required standards, indicating that the formal survey data did not fit the single-factor model. Therefore, it can be concluded that there is no significant common method bias in the formal survey data of this study.

Normality tests for univariate and multivariate data of the formal survey were conducted using AMOS 24.0. The kurtosis values for all items were well below 10, and the skewness values were within the absolute value of 3, indicating that the univariate data of the formal survey is approximately normally distributed. Therefore, the univariate data of the formal survey is approximately normally distributed. The multivariate normality test results showed that the C. R. value corresponding to the kurtosis of the multivariate data was greater than 5, indicating that the formal survey data did not meet the criteria for multivariate normal distribution. Therefore, subsequent analyses were corrected using the Bollen-Stine bootstrap method.

### 5.2 Reliability and validity tests

#### 5.2.1 Construct validity

In the CFA stage, the factor structure of the active sport tourism Experience Scape was first analyzed, followed by the development of a second-order six-dimensional factor model for the active sport tourism Experience Scape, Flow Experience, and Psychological Recovery measurement models. Before conducting the second-order confirmatory factor analysis (CFA), a first-order CFA and correlation coefficient tests between the different dimensions of the active sport tourism Experience Scape were performed. Only after the indicators met the required standards did we proceed to the second-order CFA. The first-order CFA revealed that some items had low factor loadings, so those items with insufficient loadings were removed. After the removal of the items, the results of the first-order CFA showed that the goodness of fit for the active sport tourism Experience Scape (*χ*^2^ = 139.889, *df* = 120, *χ*^2^/*df* = 1.116, CFI = 0.99, IFI = 0.99, TLI = 0.987, RMSEA = 0.024) was good. Additionally, the standardized correlation coefficients between the dimensions were all above 0.4, indicating that the model was suitable for proceeding to the second-order CFA. The second-order CFA revealed that the second-order six-dimensional factor model for the active sport tourism Experience Scape, Flow Experience, and Psychological Recovery measurement models (*χ*^2^ = 324.124, *df* = 266, *χ*^2^/*df* = 1.219, CFI = 0.979, IFI = 0.979, TLI = 0.976, RMSEA = 0.028) showed a good fit. In conclusion, the structural validity of the active sport tourism Experience Scape, Flow Experience, and Psychological Recovery demonstrated a good fit.

#### 5.2.2 Convergent validity

The Cronbach’s α coefficient was calculated using SPSS 27.0 software to measure the internal consistency of the questionnaire data. It was found that the Cronbach’s α coefficients for the overall active sport tourism Experience Scape scale, Flow Experience, Psychological Recovery, and the six subdimensions of the experience scape—social, cultural, natural, functional, sensory, and hospitality culture—were all above 0.6. The composite reliability (CR) values for each were also greater than 0.6, meeting the reliability validation standards. Convergent validity was further tested using the Average Variance Extracted (AVE). The AVE values for the dimensions of the active sport tourism Experience Scape, Flow Experience, and Psychological Recovery were all greater than 0.36, with factor loadings generally exceeding 0.5. This indicates that the convergent validity of the variable dimensions met the required standards (see Table 2).

**Table 2:** Measurement model analysis results.

Variables	Items	Factor loading	Cronbach’ α	CR	AVE
Experiencescape	Social dimension	0.929	0.899	0.913	0.637
	Functional dimension	0.698			
	Natural dimension	0.781			
	Cultural dimension	0.847			
	Sensory dimension	0.818			
	Hospitality cultural dimension	0.69			
Social dimension	The staff here (such as guides, coaches, etc.) are very friendly to me.	0.662	0.718	0.716	0.457
	The residents here are very friendly to me.	0.655			
	I get along very well with other tourists here.	0.707			
Functional dimension	The restrooms here are sufficient in number and easily accessible.	0.666	0.743	0.743	0.491
	The infrastructure here is convenient and well-developed.	0.682			
	There are relaxation areas here suitable for post-sport recovery.	0.747			
Natural dimension	The natural environment here is well-preserved.	0.703	0.707	0.706	0.445
	The natural scenery here meets my expectations.	0.64			
	The natural environment here deeply attracts me.	0.654			
Cultural dimension	The sportswear and behavior of the people here are similar to mine.	0.635	0.652	0.656	0.389
	The sports culture here is rich, with a variety of sports activities.	0.565			
	The overall cultural atmosphere here deeply attracts me.	0.669			
Sensory dimension	The tactile experiences brought by the sports activities here make me feel very comfortable.	0.671	0.675	0.681	0.519
	The overall experience here is very pleasant to my senses.	0.759			
Hospitality culture dimension	The hotel service pays attention to detail and especially shows care for sports tourists.	0.837	0.865	0.869	0.624
	The hotel offers personalized services that meet the special needs of sports tourists.	0.823			
	The overall service quality of the hotel exceeds my expectations.	0.767			
	The services provided by the hotel can meet all my needs.	0.727			
Flow experience	During the sports activity, I feel that time passes quickly.	0.653	0.687	0.694	0.432
	During the sports activity, I feel very focused.	0.598			
	When participating in sports activities, I often become fully immersed.	0.715			
Psychological recovery	After the trip, I feel relaxed and happy.	0.699	0.728	0.727	0.401
	After the trip, I cherish and love life even more.	0.59			
	After the trip, I feel refreshed.	0.604			
	After the trip, I forget about my troubles.	0.634			

#### 5.2.3 Discriminant validity

Henseler et al. (2015) confirmed that the Heterotrait-Monotrait Ratio (HTMT) developed by them effectively overcomes the limitations of traditional discriminant validity methods. When the HTMT value is less than 0.900, a good discriminant validity can be established between variables. As shown in Table 3, the HTMT values between the variables are all below 0.900, indicating that good discriminant validity exists between the variables.

**Table 3:** Discriminative validity test.

Variables	1	2	3	4	5
	EXP	FE			
FE	0.654				
PR	0.723	0.804			
	SOD	FUD	NAD	CUD	SED
FUD	0.553				
NAD	0.752	0.437			
CUD	0.716	0.797	0.54		
SED	0.738	0.492	0.714	0.686	
HCD	0.618	0.702	0.472	0.771	0.515

EXP, experiencescape; PR, psychological recovery; FE, flow experience; SOD, social dimension; FUD, functional dimension; NAD, natural dimension; CUD, cultural dimension; SED, sensory dimension; HCD, hospitality cultural dimension.

### 5.3 Hypothesis testing

To verify the influence mechanism of active sport tourism experiencescape and its six dimensions (social, functional, natural, cultural, sensory, and hospitality cultural) on psychological recovery, and to examine the mediating role of flow experience, a structural equation model (SEM) was constructed using AMOS 24.0 software with the experiencescape as the independent variable, psychological recovery as the dependent variable, and flow experience as the mediating variable (*n* = 288). The model fit indices showed a good fit, allowing for further path analysis. Overall analysis of the model indicated that active sport tourism experiencescape had a significant positive impact on psychological recovery (*β* = 0.452, *p* < 0.01), and a particularly strong positive impact on flow experience (*β* = 0.731, *p* < 0.001). Moreover, flow experience also had a significant positive effect on psychological recovery (*β* = 0.478, *p* < 0.01). This suggests that the experiencescape not only directly promotes psychological recovery but also indirectly enhances psychological recovery by increasing flow experience.

Further path analysis revealed significant differences in the direct effects of the various dimensions of the experiencescape on psychological recovery. The social dimension (*β* = 0.518, *p* < 0.05), natural dimension (*β* = 0.327, *p* < 0.05), sensory dimension (*β* = 0.346, *p* < 0.001), and hospitality cultural dimension (*β* = 0.214, *p* < 0.01) had significant direct effects on psychological recovery, while the functional dimension (*β* = 0.108, *p* > 0.05) and cultural dimension (*β* = 0.217, *p* > 0.05) did not have significant direct effects. Therefore, hypotheses H1a, H2a, H2b, and H3 were supported, while H1b was not supported (see Table 4). The above results indicate that the social dimension had the most prominent impact. Its significant direct effect suggests that positive interactions with others (such as fellow tourists, coaches, or guides) during participation in the experiencescape can effectively enhance psychological recovery. At the same time, the significant impact of the natural and sensory dimensions on psychological recovery further underscores the importance of environmental factors. The significant effect of the natural dimension indicates that the experience of natural landscapes during outdoor activities helps release psychological stress and regulate emotions, while the sensory dimension highlights the important role of diverse sensory stimuli (such as visual, auditory, and tactile experiences) in psychological recovery.

**Table 4:** Direct effect test results.

Hypothesized path	Beta	SE	95%CI	*P*	Status
H1a: EXP → PR	0.452	0.155	(0.132, 0.732)	0.008	Supported
H3: FE → PR	0.478	0.149	(0.202, 0.780)	0.001	Supported
H1b: SOD→PR	0.518	0.215	(0.099, 0.890)	0.021	Supported
H1b: FUD → PR	0.108	0.098	(−0.085, 0.301)	0.245	Unsupported
H1b: NAD → PR	0.327	0.137	(0.013, 0.559)	0.04	Supported
H1b: CUD → PR	0.217	0.118	(−0.013, 0.441)	0.063	Unsupported
H1b: SED → PR	0.346	0.14	(0.055, 0.610)	0	Supported
H1b: HCD → PR	0.214	0.08	(0.065, 0.382)	0.004	Supported
H2a: EXP → FE	0.731	0.064	(0.587, 0.839)	0	Supported
H2b: SOD→FE	0.779	0.076	(0.608, 0.905)	0	Supported
H2b: FUD → FE	0.372	0.11	(0.176, 0.595)	0.001	Supported
H2b: NAD → FE	0.63	0.08	(0.468, 0.778)	0	Supported
H2b: CUD → FE	0.54	0.079	(0.386, 0.690)	0	Supported
H2b: SED → FE	0.629	0.084	(0.455, 0.787)	0.021	Supported
H2b: HCD → FE	0.357	0.072	(0.214, 0.494)	0	Supported

The mediation effect was tested using the Bootstrap method, with 5,000 resamples and a 95% confidence interval, to systematically assess the indirect impact of the experiencescape in active sport tourism on psychological recovery through flow experience. The test results show that the experiencescape has a significant indirect effect on psychological recovery through flow experience (*β* = 0.349, *p* < 0.01), and there are significant differences in the indirect effects across different dimensions. Hypotheses H4b and H4a are supported (see Table 5). The social dimension shows a significant indirect effect on psychological recovery through flow experience (*β* = 0.316, *p* < 0.05), but its direct effect is also significant, indicating that flow experience only plays a partial mediating role in this path. This result suggests that interaction with others not only directly enhances psychological recovery but also further promotes psychological recovery by enhancing flow experience. Similarly, the natural dimension (*β* = 0.377, *p* < 0.001), sensory dimension (*β* = 0.369, *p* < 0.001), and hospitality cultural dimension (*β* = 0.257, *p* < 0.001) also exhibited significant partial mediation effects. This indicates that the attractiveness of the natural environment, sensory immersion experiences, and the uniqueness of hospitality culture not only directly enhance psychological recovery but also amplify this positive effect by enhancing flow experience. On the other hand, the functional dimension (*β* = 0.283, *p* < 0.01) and cultural dimension (*β* = 0.368, *p* < 0.001) show significant indirect effects on psychological recovery through flow experience, but their direct effects are not significant. This suggests that in these two dimensions, flow experience plays a full mediating role, acting as a crucial bridge. The impact of the functional dimension may be reflected in the professionalism of activity organization and the smoothness of the experience, while the cultural dimension is more reflected in the attractiveness of cultural content and the depth of the experiential feeling. These results emphasize the central role of flow experience in the pathway through which the functional and cultural dimensions affect psychological recovery.

**Table 5:** Mediation effect test results.

Hypothesized path	Beta	SE	95%CI	*P*	Status
H4a: EXP → FE → PR	0.349	0.12	(0.160, 0.625)	0.001	Supported
H4b: SOD→FE → PR	0.316	0.193	(0.020, 0.729)	0.039	Supported
H4b: FUD → FE → PR	0.283	0.104	(0.125, 0.533)	0.001	Supported
H4b: NAD → FE → PR	0.377	0.099	(0.235, 0.632)	0	Supported
H4b: CUD → FE → PR	0.368	0.098	(0.222, 0.605)	0	Supported
H4b: SED → FE → PR	0.369	0.09	(0.228, 0.597)	0	Supported
H4b: HCD → FE → PR	0.257	0.065	(0.147, 0.404)	0	Supported

## 6 Discussion

### 6.1 Conclusion

This study introduces the experiencescape theory into the context of active sport tourism, exploring the impact mechanism of the experiencescape in active sport tourism on psychological recovery and focusing on the mediating role of flow experience. The main findings are as follows:

First, the experiencescape in active sport tourism consists of six dimensions: social, natural, functional, cultural, sensory, and hospitality culture. This classification not only extends Pizam and Tasci (2019) generalized definition of experiencescape but also integrates the contextual characteristics of sports tourism, enriching and expanding the content dimensions of experiencescape. This approach fills the gap left by previous research in the field of sports tourism. Compared to Zhang and Xu (2023) more general classification of tourism experiencescape into physical, natural, social, and social-symbolic dimensions, this study further refines the dimensions of experiencescape, based on the practical context of active sport tourism, making them more aligned with the unique attributes of sports tourism activities. At the same time, compared with the multi-dimensional classifications proposed by Liu and Lin (2024) for rural tourism and Zong et al. (2023) for Hanfu tourism, this study emphasizes the highly dynamic and contextual characteristics of the experiencescape in active sport tourism. For example, the inclusion of the hospitality culture dimension reflects the potential impact of accommodation settings on the overall visitor experience in active sport tourism and provides new insights for future research on other types of tourism experiencescapes. Moreover, the experiencescape model developed in this study has strong applicability and specificity, differing from Li and Zhang (2022) restrictive definitions of thematic atmosphere and social participation elements in the experiencescape of red tourism.

Second, the experiencescape in active sport tourism not only has a significant direct positive impact on flow experience and psychological recovery but also indirectly influences psychological recovery through the partial mediating effect of flow experience. This result is consistent with the view of Tasci and Pizam (2020), providing empirical support for examining the multidimensional integrated effects of experiencescape on enhancing visitors’ psychological experience and recovery outcomes. Furthermore, the functional and cultural dimensions do not have a direct effect on psychological recovery; their effects need to be mediated through flow experience. This suggests that the functional dimension enhances visitors’ focus and sense of psychological flow by offering appropriate challenges and quality facilities, while the cultural dimension promotes social and cultural identity and indirectly influences psychological recovery through rich cultural activities and emotional resonance. This result deepens the understanding of the mechanisms of the functional and cultural dimensions, expanding the research boundaries of the experiencescape theory.

Third, flow experience, as the key bridge between experiencescape and psychological recovery, exhibits significant differences in the influence paths of different dimensions on flow experience and psychological recovery. This study clearly identifies for the first time the differential mediation effects of flow experience across different dimensions of experiencescape. In the social, natural, sensory, and hospitality cultural dimensions, flow experience serves as a partial mediator, indicating that these dimensions have both direct effects on psychological recovery and indirect effects through flow experience. In contrast, the functional and cultural dimensions rely entirely on the mediation mechanism of flow experience, with a more complex underlying mechanism. This finding enriches the application of flow experience theory in complex tourism scenarios and provides new theoretical and practical foundations for future experiencescape design and psychological recovery interventions.

### 6.2 Theoretical implications

First, this study extends the application of experiencescape theory, providing empirical support for its applicability in the field of sports tourism. The study confirms that experiencescape, as an overall variable, significantly promotes psychological recovery and further enhances this effect through flow experience. This finding enriches the connotation of experiencescape theory and deepens its application in dynamic and highly interactive contexts like sports tourism.

Second, this study reveals the differentiated impact mechanisms of the multi-dimensional structure of experiencescape on psychological recovery. The study finds that the social, natural, sensory, and hospitality cultural dimensions have direct effects on psychological recovery, while the functional and cultural dimensions exert their effects indirectly through the complete mediation of flow experience. This provides a more detailed dimensional analysis for experiencescape theory and offers practical guidance for scene design, i.e., prioritizing the reinforcement of dimensions with direct recovery effects while also enhancing the role of functionality and culture through flow experience.

Third, this study deepens the theoretical role of flow experience as a mediating variable. The results show that flow experience has significantly differentiated mediating effects across different dimensions of experiencescape, with some dimensions (e.g., social dimension) exhibiting partial mediation, while others (e.g., functional dimension) demonstrate complete mediation. This further validates the central role of flow experience in the psychological recovery mechanism and refines its specific pathways of influence.

Finally, this study innovatively combines experiencescape with the S-O-R model, revealing the mechanisms through which different dimensions of experiencescape influence psychological recovery via flow experience. This provides theoretical support for the dynamic and contextual development of the S-O-R model.

### 6.3 Management implications

First, optimize social interaction scenes to enhance psychological recovery. The study shows that the social dimension not only directly promotes psychological recovery but also strengthens its effect through flow experience. Therefore, destination and event planners should focus on designing activities that promote interaction between tourists, between tourists and service staff, and between tourists and local residents, such as team-building outdoor projects, deep cultural exchange activities, and experiential projects co-created with locals.

Second, strengthen the direct influence of natural, sensory, and hospitality cultural elements. The study finds that these dimensions have a direct effect on psychological recovery. Therefore, in destination development, natural resources should be fully utilized to design activities that integrate with the natural environment, such as hiking, trekking, and diving, while also focusing on the diversity of sensory experiences, such as unique visual landscapes, sound experiences, and taste stimuli. In addition, as a key part of the tourist’s stay, hospitality culture should integrate local characteristics and cultural elements to provide an immersive experience for tourists.

Third, enhance the guiding role of flow experience in functional and cultural dimensions. The study shows that the psychological recovery effects of the functional and cultural dimensions rely on the complete mediation of flow experience. Therefore, planners should carefully design activity content and scene settings to enhance the sense of challenge and immersion for tourists. For example, setting outdoor sports projects with appropriate levels of difficulty or offering activities with in-depth cultural experiences can help tourists quickly enter a flow state, thereby indirectly improving their psychological recovery.

Fourth, focus on the overall optimization design of experiencescape. The study validates the significant promoting effect of experiencescape on psychological recovery, indicating that when designing sports tourism products, the synergistic effects of various dimensions should be considered. Destinations should integrate resources such as social interaction, natural landscapes, functional activities, and cultural displays to form multi-dimensional, comprehensive experiencescapes, thereby meeting the diverse needs of tourists and enhancing the overall value of the experience.

### 6.4 Limitations and research directions

Although this study has achieved certain results in exploring the impact of active sport tourism experiencescapes on tourists’ psychological recovery, there are still some limitations, and future research can further improve upon these findings.

First, this study primarily used cross-sectional data to analyze the impact of active sport tourism experiencescapes on tourists’ psychological recovery. While this design can reveal correlations between variables, it cannot determine the directionality of causal relationships. Future research could further verify the causal mechanisms of active sport tourism experiencescapes on psychological recovery using longitudinal or experimental designs, thus enhancing the robustness of the research conclusions. Second, this study used flow experience as a mediating variable to explore the mechanism by which active sport tourism experiencescapes affect tourists’ psychological recovery. However, the factors influencing tourists’ psychological recovery may be multiple, and in addition to flow experience, they may include other psychological mechanisms such as emotion regulation and social support. Future research could introduce more mediating or moderating variables, constructing more complex models to comprehensively reveal the formation mechanism of tourists’ psychological recovery. Additionally, the self-report data used in this study may be affected by social desirability bias and memory recall bias. Future research could combine physiological indicators or behavioral data, adopting a multi-source data approach to further enhance the accuracy and scientific rigor of the study. Finally, with the continuous development of active sport tourism, emerging forms of tourism and technologies (such as virtual reality experiences, digital guides, etc.) may have new impacts on tourists’ experiences and psychological recovery. Future research could focus on these emerging trends, adding a technological dimension to experiencescape, exploring their effects on tourism experience and psychological recovery, and providing cutting-edge theoretical support for the innovation and development of sports tourism.

## Data Availability

The original contributions presented in the study are included in the article/supplementary material, further inquiries can be directed to the corresponding author.
